# Leptin Production by Encapsulated Adipocytes Increases Brown Fat, Decreases Resistin, and Improves Glucose Intolerance in Obese Mice

**DOI:** 10.1371/journal.pone.0153198

**Published:** 2016-04-07

**Authors:** David J. DiSilvestro, Emiliano Melgar-Bermudez, Rumana Yasmeen, Paolo Fadda, L. James Lee, Anuradha Kalyanasundaram, Chen L. Gilor, Ouliana Ziouzenkova

**Affiliations:** 1 Department of Human Sciences, The Ohio State University, Columbus, Ohio, 43210, United States of America; 2 Genomics Shared Resource, Comprehensive Cancer Center, The Ohio State University, Columbus, Ohio, 43210, United States of America; 3 NSF Nanoscale Science and Engineering Center for Affordable Nanoengineering of Polymeric Biomedical Devices, The Ohio State University, Columbus, Ohio, United States of America; 4 Department of Physiology and Cell Biology, Davis Heart and Lung Research Institute, The Ohio State University, Columbus, Ohio, 43210, United States of America; 5 Veterinary Clinical Sciences, The Ohio State University, Columbus, Ohio, 43210, United States of America; State University of Rio de Janeiro, Biomedical Center, Institute of Biology, BRAZIL

## Abstract

The neuroendocrine effects of leptin on metabolism hold promise to be translated into a complementary therapy to traditional insulin therapy for diabetes and obesity. However, injections of leptin can provoke inflammation. We tested the effects of leptin, produced in the physiological adipocyte location, on metabolism in mouse models of genetic and dietary obesity. We generated 3T3-L1 adipocytes constitutively secreting leptin and encapsulated them in a poly-L-lysine membrane, which protects the cells from immune rejection. *Ob/ob* mice (OB) were injected with capsules containing no cells (empty, OB^[Emp]^), adipocytes (OB^[3T3]^), or adipocytes overexpressing leptin (OB^[*Lep*]^) into both visceral fat depots. Leptin was found in the plasma of OB^[*Lep*]^, but not OB^[Emp]^ and OB^[3T3]^ mice at the end of treatment (72 days). The OB^[*Lep*]^ and OB^[3T3]^ mice have transiently suppressed appetite and weight loss compared to OB^[Emp]^. Only OB^[*Lep*]^ mice have greater brown fat mass, metabolic rate, and reduced resistin plasma levels compared to OB^[Emp]^. Glucose tolerance was markedly better in OB^[*Lep*]^ *vs*. OB^[Emp]^ and OB^[3T3]^ mice as well as in wild type mice with high-fat diet-induced obesity and insulin resistance treated with encapsulated leptin-producing adipocytes. Our proof-of-principle study provides evidence of long-term improvement of glucose tolerance with encapsulated adipocytes producing leptin.

## Introduction

Obesity affects 150 million people worldwide and is characterized as pandemic disease [1, 2]. In North America, European Union, China, and other countries, obesity is considered to be a major risk factor for up to 70–90% of the adult cases of type II diabetes mellitus (T2DM) [1–3]. The treatment of diabetes with insulin induces accumulation of lipids in adipose, muscle, and other peripheral tissues [4]. These effects reduce the efficacy of insulin therapy and are responsible for the detrimental side effects increasing cardiovascular mortality in patients with diabetes [5]. There is a critical need to develop therapies to treat obesity and diabetes that simultaneously improve lipid and glucose homeostasis [4].

The adipokine leptin has been recently considered as a hormone candidate for managing diabetes in the absence or presence of insulin diabetes therapy [6]. Leptin mediates its therapeutic effects on energy homeostasis through the hypothalamus and selected action on specific neurons [7–14]. Leptin induces hypothalamic secretion of the appetite suppressing corticotrophin-releasing hormone [11]. Leptin stimulation releases sympathetic neurotransmitters, norepinephrine and epinephrine, that increase activation of lipolysis and thermogenesis in white adipose tissue (WAT) and brown adipose tissues (BAT) [14, 15]. Leptin works synergistically with insulin to promote thermogenesis in WAT and increasing energy expenditure through hypothalamic neurons [16]. Cumulatively, these effects, after leptin administration, reduce obesity in mouse models of obesity and in human patients with homozygous *Lep* mutations or congenital *Lep* deficiency [17–19]. Leptin’s simulation of the hypothalamus increases glucose uptake in BAT, heart muscle, and skeletal muscle, but not WAT [12, 13]. Leptin and insulin also synergize to increase glucose uptake in these tissues, in part via suppression of glucagon [12]. These responses make leptin a promising biological target for a complementary therapy to the traditional insulin treatments for diabetes and obesity.

A primary model for the development of leptin therapies is the *ob/ob* mouse (*Lep*^*ob*^, or *ob*), a homozygous mutant in the leptin (*Lep or ob* gene) gene. In the absence of functioning leptin, the *ob/ob* mouse rapidly develops obesity and hyperglycemic conditions similar to T2DM [6, 20–22]. Reintroduction of leptin by injection of exogenous recombinant leptin, adenovirus transduction restoring leptin expression in tissues, or transplantation of tissues producing leptin attenuate weight gain, improve glucose tolerance, decrease appetite, and increase metabolic rate in *ob/ob* mice [6, 23–26]. However, these methods of leptin delivery cannot be directly translated into therapies for humans. Leptin replacement therapy is necessary in patients with homozygous *Lep* mutations or acquired leptin dysfunction that produces congenital and acquired generalized lipodystrophy [17–19]. Treating these patients with recombinant leptin through injections produces short lasting effects and requires repetitive doses. The supra-physiological increase in leptin in the circulation followed by regular injections is associated with serious side effects [27]. One study reports that endogenous production of leptin works better than insulin injections [28]. However, there are limitations on how to safely increase endogenous production of leptin. Increasing endogenous production by transduction using adenoviruses carrying a functioning *Lep* gene [28, 29] or by the transplantation of tissues or cells expressing functional leptin [26, 30, 31] pose risks of infection, immune rejection of the transplant, or potential genetic viral contamination that could be hazardous to human health. Transplantation models also require drugs to suppress the host’s immune system. To improve transplant survival, Oosman et al, suspended leptin-producing intestinal cells in alginate beads; however, this treatment also requires immunosuppression [26].

Recently, we employed a nanotechnology to develop a procedure of adipocyte microencapsulation [32] for the transplantation of genetically engineered cells into adipose tissue without immune rejection [33]. The encapsulating poly-L-lysine generates a nanoporous membrane that protects the encapsulated cells from the host’s immune system as well as allows small molecules, like hormones, to diffuse out of the capsule. Here we show that treatment with encapsulated leptin-producing adipocytes improves glucose tolerance in genetic and diet-induced mouse models of obesity. Encapsulated leptin-producing adipocytes also reveal novel aspects of leptin signaling including suppression of resistin in *ob/ob* mice.

## Results

### Encapsulated adipocytes overexpressing *Lep* secrete leptin *in vivo* and *in vitro*

**Fig 1:**
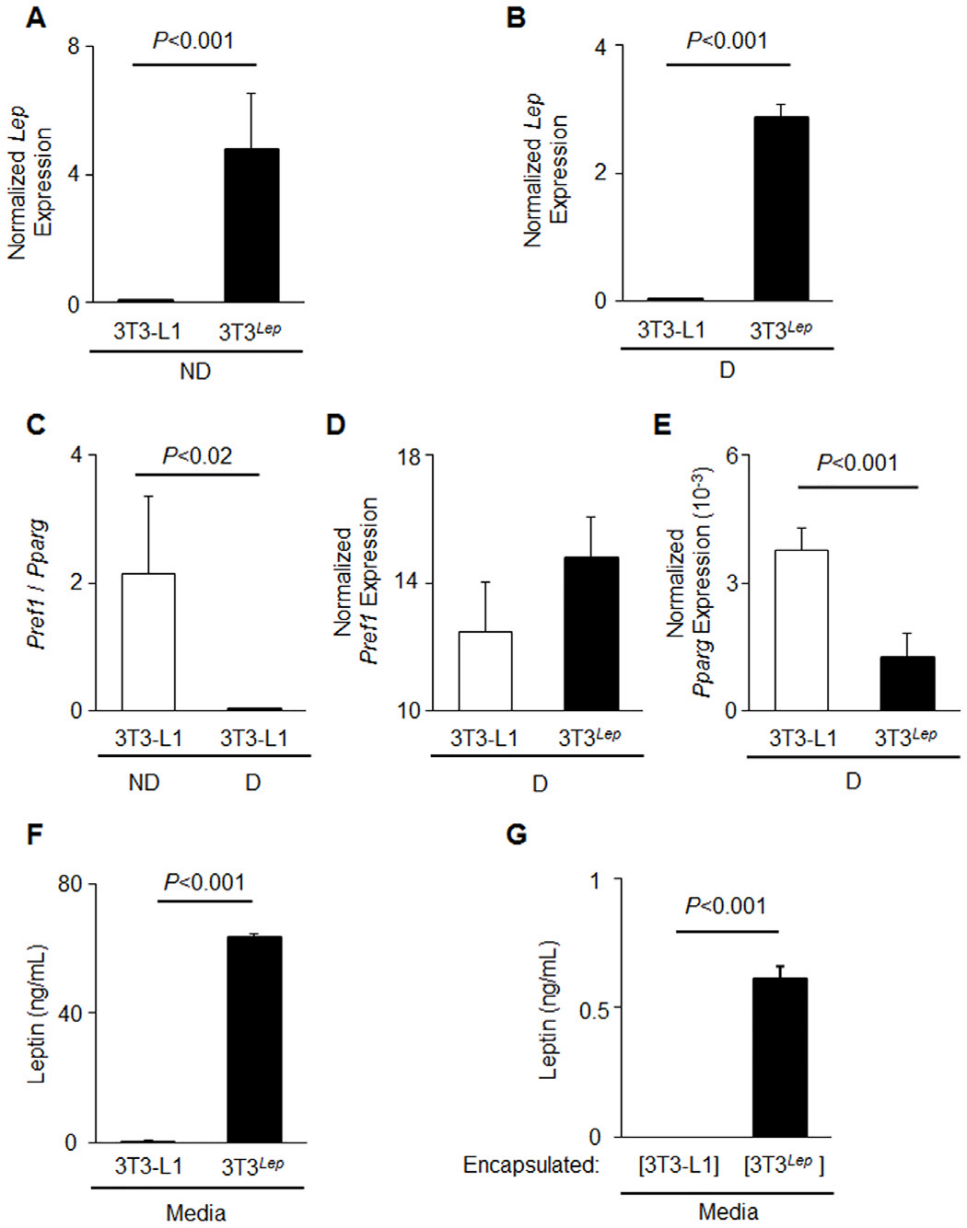
Engineered 3T3-L1 adipocytes express and constitutively secrete leptin. 3T3-L1 cells were stably transduced to overexpress *Lep* (3T3^*Lep*^) (A, B) *Lep* expression in non-differentiated preadipocytes (ND = non-differentiated) (A) and differentiated preadipocytes (D = differentiated) (B) is shown for 3T3-L1 (white bar) and 3T3^*Lep*^ (black bar) (both n = 4, *P*<0.001). All gene expression was measured by qRT-PCR and normalized to TATA box binding protein (TBP). (C) The ratio of *Pref1* to *Pparg* expression non-differentiated (ND, white bar) and differentiated (D, black bar) and 3T3-L1 (white bar) (n = 3, *P<*0.02). (D, E) *Pref1* (D) and *Pparg* (E) expression in differentiated (both n = 4) 3T3-L1 (white bar) and 3T3^*Lep*^ (black bar, *P*<0.001). (F) A mouse leptin ELISA was performed on media collected from confluent differentiated 3T3-L1 (white bar) and 3T3^*Lep*^ (black bar) adipocytes that were incubated in this media for 48 hours (n = 4, *P*<0.001). (G) Leptin concentration was measured by ELISA in media collected from a monolayer of encapsulated 3T3-L1 ([3T3-L1], white bar) and 3T3^*Lep*^ ([3T3^*Lep*^], black bar) adipocytes cultured overnight. All data are expressed as means ± SD (n = 4, *P*<0.001).

The generated *Lep* (3T3^*Lep*^) preadipocytes and adipocytes expressed approximately 90,000 times greater levels of *Lep* compared to 3T3-L1 preadipocytes before (Fig 1A) and after differentiation (Fig 1B). We validated adipogenesis using markers of preadipocytes, *Pref1*, and differentiated adipocytes, *Pparg* [34]. A ratio of expressed *Pref1* to *Pparg* was 98.4% lower in differentiated *vs*. non-differentiated 3T3-L1 cells (*P*<0.02) (Fig 1C). Both 3T3-L1 and 3T3^*Lep*^ adipocytes differentiated for six days expressed similar levels of *Pref1* (Fig 1D, n = 4, *P<*0.06). *Pparg* expression was 33% lower than in differentiated 3T3^*Lep*^ adipocytes (*P*<0.001) (Fig 1E). To determine if *Lep* was translated and secreted, the supernatant media was analyzed for secreted leptin in differentiated 3T3-L1 and 3T3^*Lep*^adipocytes. 3T3^*Lep*^, but not 3T3-L1, secreted leptin into media (63.4 ± 13 ng/mL vs. 0.01 ± 0.01 ng/mL, n = 4, *P<*0.001) (Fig 1F). To test the release of leptin from encapsulated preadipocytes, 3T3-L1 or 3T3^*Lep*^ were encapsulated in poly-L-lysine (described as [3T3-L1] and [3T3^*Lep*^], where brackets indicate encapsulation). The detailed procedure and videoprotocol is described in [32]. After encapsulated cells were cultured overnight, media was collected and analyzed for secreted leptin. Encapsulation did not alter the leptin secretion by 3T3-L1 and 3T3^*Lep*^ adipocytes, [3T3^*Lep*^], but not [3T3-L1], produced leptin (Fig 1G). The concentration of leptin produced by encapsulated adipocytes was less than those of cultured preadipocytes (0.6 ng/mL vs. 63.4 ng/mL) due to the smaller number of encapsulated cells in the well and shorter incubation time (Fig 1F vs. Fig 1G). Thus, encapsulation permits leptin production and secretion from [3T3^*Lep*^] cells *in vitro*.

**Fig 2:**
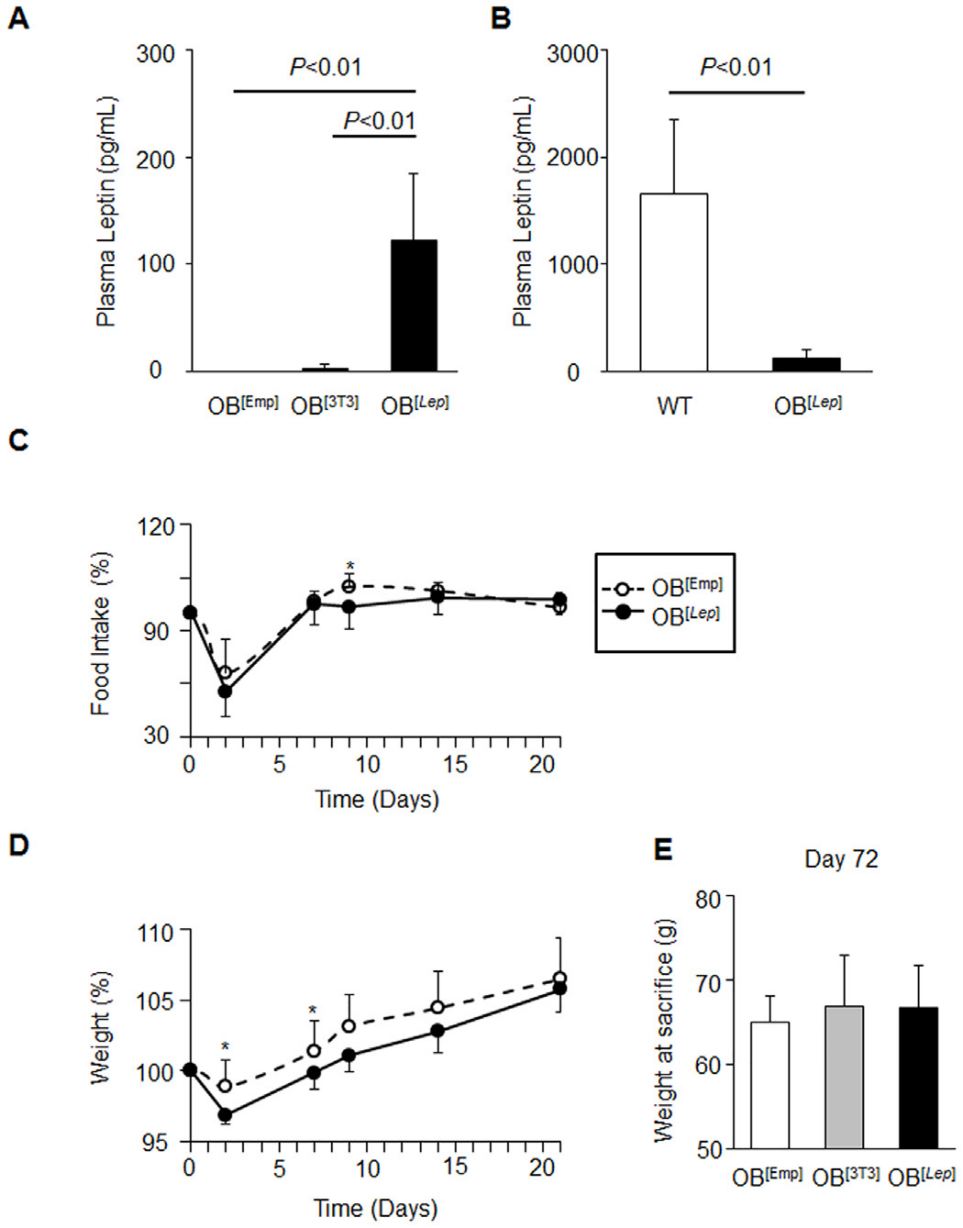
Treatment with leptin-producing encapsulated adipocytes increases plasma leptin and transiently influences food intake and weight. O*b/ob* mice (n = 7 per group) were injected with encapsulated acellular capsules (OB^[Emp]^), encapsulated 3T3-L1 preadipocytes (OB^[3T3]^), and encapsulated 3T3^*Lep*^ (OB^[*Lep*]^) in both visceral fat pads (total 0.6*10^6^ adipocytes for each cell type). (A) Leptin concentrations in plasma from OB^[Emp]^ (n = 4), OB^[3T3]^ (n = 4), and OB^[*Lep*]^ (n = 7) mice. The leptin ELISA was performed on non-hemolyzed plasma samples. All data are expressed as means ± SD. Significant differences are shown with a black bar and determined by one-way ANOVA followed by Tukey’s post hoc test (*P*<0.01). (B) Comparison of leptin concentrations in plasma from OB^[*Lep*]^ (n = 7) and WT mice fed a high fat (HF) diet for 60 days (n = 5). Leptin was analyzed by ELISA, (*P*<0.01, Student’s t-test). (C) Average food intake kinetics in OB^[Emp]^ (open circles, n = 6) and OB^[*Lep*]^ (closed circles, n = 7) mice are shown as percent of intake prior to injection. Asterisks show *P<*0.05, Student’s t-test. Data for OB^[3T3]^ mice is in S1 Fig. (D) Average weight kinetics in same groups of mice. Weight is shown as percent of weight prior to injection (g/g prior to injection x 100). Asterisks show *P<*0.05, Student’s t-test. (E) Weights at sacrifice (72 days post-treatment, mean ± SD) in same group of mice.

To test survival and production of leptin by [3T3^*Lep*^] adipocytes *in vivo*, we injected three groups of *ob/ob* mice with encapsulated acellular capsules (OB^[Emp]^), encapsulated 3T3-L1 preadipocytes (OB^[3T3]^), and encapsulated 3T3^*Lep*^ (OB^[*Lep*]^) in both visceral fat pads. Average plasma leptin concentrations were measured 72 days post capsule injection in OB^[Emp]^, OB^[3T3]^, and OB^[*Lep*]^ to be 0 ± 0, 2.59 ± 4.5, and 122 ± 63 pg/mL, respectively (Fig 2A). Plasma leptin levels were statistically greater in OB^[*Lep*]^ than the other two groups (*P*<0.01). However, OB^[*Lep*]^ had lower plasma leptin levels when compared to WT mice fed a HF diet (7.4%, 0.22 ± 0.62 ng/mL vs. 1.66 ± 0.08 ng/mL) (Fig 2B). Therefore, encapsulated engineered adipocytes engrafted in their physiological location in adipose tissues can increase plasma levels of leptin for prolonged periods of time.

### Leptin treatment by encapsulated adipocytes increases BAT and metabolic rate

We recorded kinetics of food intake and weight gain for OB^[Emp]^, OB^[3T3]^, and OB^[*Lep*]^ 14 days before (S1A and S1B Fig) and 35 days after 21 days of treatment (Fig 2C and 2D) and weight at the end of treatment (Fig 2E). Given the variability of individual metabolic responses in mice, all food intake and weight data were normalized to the initial food intake (100%) or weight (100%) in each mouse before treatment. The treatment with encapsulated adipocytes transiently suppressed appetite in OB^[*Lep*]^ compared to OB^[Emp]^ mice. Food intake in OB^*Lep*^ group was statistically lower than in OB^[Emp]^ mice on day 9 post injection (Fig 2D). The suppression of food intake coincided with transiently decreased weight in OB^[*Lep*]^ mice compared to OB^[Emp]^ on days 2 and 7 post injection (Fig 2C). Treatment with encapsulated 3T3-L1 cells also attenuated food intake and weight gain when compared to OB^[Emp]^ mice (S1C and S1D Fig). Both food intake and weight were not different between OB^[*Lep*]^ and OB^[3T3]^ groups. Thus, treatment with encapsulated 3T3-L1 adipocytes with or without engineered secretion of leptin from visceral location produced only minor and transient effects on appetite and weight. To determine if these effects were due to an immune response, IL-6 was measured in plasma and the VF of these mice. IL-6 was below detection levels in plasma and OB^[Emp]^, OB^[3T3]^, and OB^[*Lep*]^ had statistically the same levels of IL-6 in the VF (S2A Fig).

**Fig 3:**
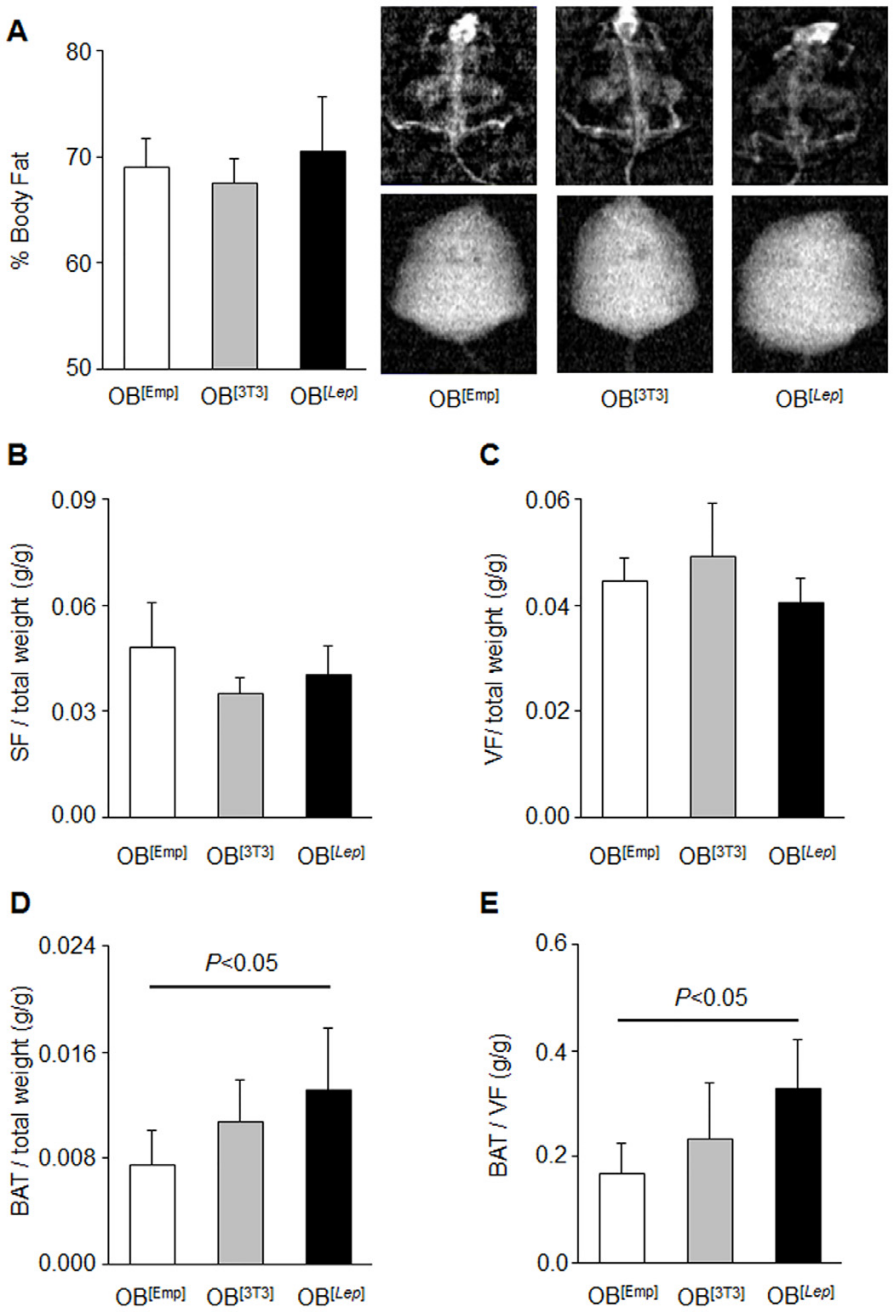
Treatment with leptin-producing encapsulated adipocytes increases BAT but not WAT weight. (A) Average percent body fat for OB^[Emp]^ (white bar, n = 5), OB^[3T3]^ (gray bar, n = 5), and OB^[*Lep*]^ (black bar, n = 7) was measured using DEXA. Representative DEXA images are shown for each group. (B-D) Average normalized weight of subcutaneous fat (SF, B), visceral (VF, C), and brown (BAT, D) to body weight in same mouse groups. (E) The average ratio of BAT to VF in same mouse groups. All data are represented as mean ± SD. Significant differences are shown by black bar and determined by one-way ANOVA followed by Tukey’s test.

At the end of study we measured percent body fat measured by DEXA and weight of dissected subcutaneous (SF), visceral (VF), and brown adipose fat (BAT) pads (Fig 3). Adipose tissues were dissected as described in Yasmeen *et al* [35]. The percent of body fat measured by DEXA (Fig 3A) as well as the weight of dissected SF (Fig 3B) and VF (Fig 3C), and liver weight (S1E Fig) were not altered by any treatment in all mouse groups. In contrast, BAT mass was increased in OB^[*Lep*]^ mice. OB^[*Lep*]^ had 175% greater BAT weight, when compared to OB^[Emp]^ (Fig 3D). This increase in BAT mass was not associated with increased protein levels of UCP1 or PGC-1α (S2B and S2D Fig). The significant increase in the BAT to VF ratio in OB^[*Lep*]^ *vs*. OB^[Emp]^ mice groups (Fig 3E) was expected to increase metabolic rate in the leptin-treated group.

**Fig 4:**
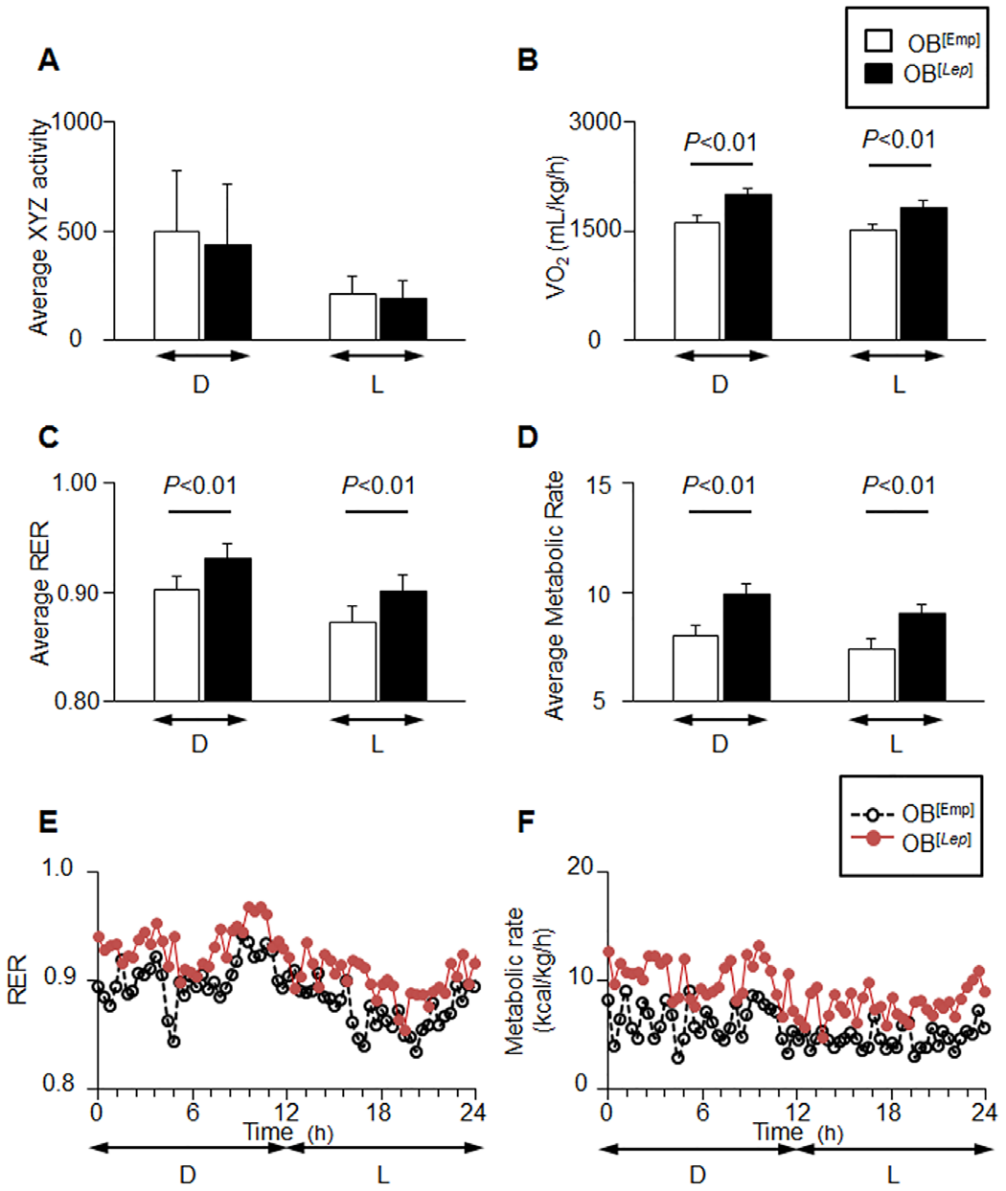
Leptin increases metabolic rate and raises RER. (A-D) Metabolic measurements were performed in OB^[Emp]^ (white bar, n = 5) and OB^[*Lep*]^ (black bar, n = 6) mouse groups in CLAMS metabolic cages at 28 days post injection. Metabolic data for OB^[3T3]^ group are shown in S3 Fig. The average (mean ± SD) x, y, and z activity (A), average VO_2_ (mL/kg/h) (B), respiratory exchange ratio (RER) (C), and average metabolic rate (kcal/h/kg) (D) are shown for both the dark (D) and light (L) cycles. (E, F) Kinetic data for RER (E) and metabolic rate (kcal/h/kg) (F) are shown as mean for each time point in OB^[Emp]^ (open black circles) and OB^[*Lep*]^ (filled red circles) groups. Significant differences are shown by black bar and determined by one-way ANOVA followed by Tukey’s test.

We tested the differences in metabolic parameters between OB^[Emp]^, OB^[3T3]^, and OB^[*Lep*]^ mouse groups using metabolic cages. Activity did not differ between groups (Fig 4A). OB^[*Lep*]^ had a greater VO_2_ (mL/kg/h) of 2000 ± 88 for dark cycle and 1822 ± 92 for light cycle than OB^Emp^ (1619 ± 113 and 1501 ± 122). The VO_2_ for OB^[*Lep*]^ mice was greater than OB^[3T3]^ group during the dark cycle (2000 ± 88 vs. 1875 ± 114) but not the light cycle (S3B Fig). OB^[*Lep*]^ group had a greater respiratory exchange ratio (RER) of 0.93 ± 0.01 for the dark cycle and 0.90 ± 0.01 for the light cycle than OB^Emp^ (0.90 ± 0.01 and 0.87 ± 0.01) and OB^[3T3]^ (0.91 ± 0.01 and 0.88 ± 0.01) for both the dark and light cycles (Fig 4C, 4E and S3C–S3E Fig) suggesting that glucose utilization is higher in OB^[*Lep*]^ compared to the other groups. The OB^[*Lep*]^ had a greater average metabolic rate of 9.89 ± 0.47 and 8.99 ± 0.46 kcal/h/kg for the dark and light cycles when compared to 7.99 ± 0.57 and 7.36 ± 0.62 kcal/h/kg for OB^[Emp]^ (*P*<0.001) (Fig 4D and 4F). OB^[*Lep*]^ had a higher metabolic rate than OB^[3T3]^ for the dark cycle (9.89 ± 0.47 vs 9.31 ± 0.66, *P*<0.005) but not the light cycle (8.99 ± 0.46 vs. 8.75 ± 0.60, *P*<0.16) (S3D–S3F Fig).

### Leptin treatment by encapsulated adipocytes improves glucose tolerance and resistin

**Fig 5:**
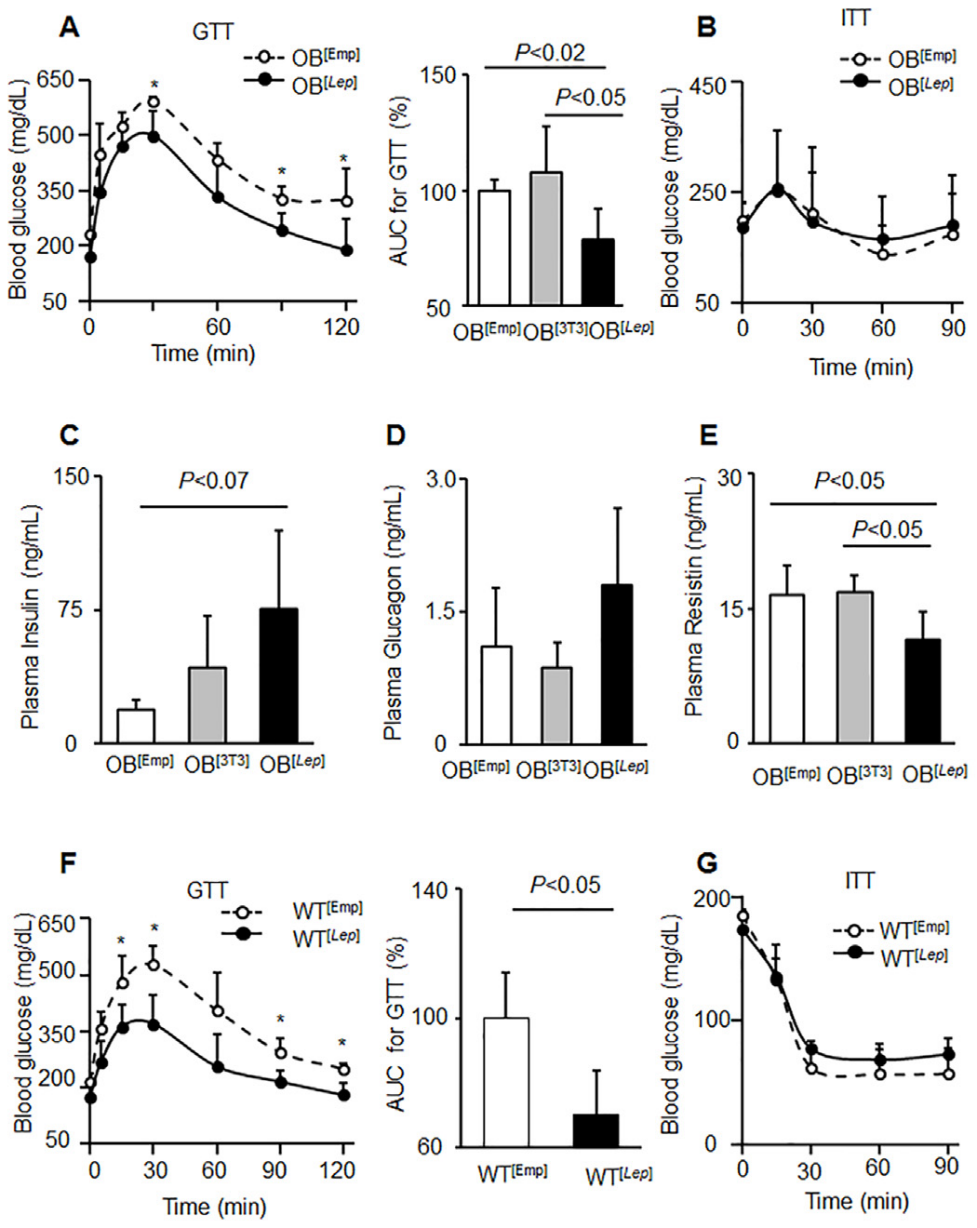
Leptin improves glucose tolerance and reduces resistin. (A,B) Glucose tolerance tests (GTT) (A) or insulin tolerance tests (ITT) (B) were performed in fasting overnight mice OB^[Emp]^ (open circles, n = 5) and OB^[*Lep*]^ (filled circles, n = 5). GTT and ITT tests for OB^[3T3]^ group are shown in S4 Fig. Average blood glucose (mg/dL) concentrations are displayed for each time point. Asterisks indicate significant differences *P*<0.05, Student’s t-test. The area under the curve (AUC) for each GTT was calculated using a trapezoidal approximation and displayed as percent of the control OB^[Emp]^ group (100%). (C-E) Plasma insulin (C), glucagon (D), and resistin (E) concentrations were measured by ELISA in OB^[Emp]^ (white bar, n = 4), OB^[3T3]^ (gray bar, n = 3), and OB^[*Lep*]^ (black bar, n = 7). Statistical difference was examined by one-way ANOVA followed by Tukey’s post hoc test. (F, G) WT mice with diet-induced obesity (Study 2) were injected with encapsulated acellular capsules (WT^[Emp]^) and encapsulated 3T3^*Lep*^ (WT^[*Lep*]^) in both visceral fat pads (total 0.6*10^6^ adipocytes for each cell type). GTT (F) or ITT (G) were performed in fasting overnight mice WT^[Emp]^ (open circles, n = 3) and WT^[*Lep*]^ (filled circles, n = 5). All data are represented as mean ± SD.

To access leptin’s role in glucose metabolism, we performed glucose tolerance and insulin tests in all studied groups. The glucose tolerance test (GTT) responses in OB^[Emp]^ and OB^[3T3]^ mice were similar (S4A and S4B Fig). OB^[*Lep*]^ mice (78 *±* 14%) were more sensitive to glucose than OB^[Emp]^ (100 ± 5%) and OB^[3T3]^ (108 ± 20%) as measured by the AUC for GTT (Fig 5B). The leptin treatment in OB^[*Lep*]^ group resulted in significantly improved glucose tolerance compared to OB^[Emp]^ and OB^[3T3]^ mice (Fig 5A, 5B and S4C, S4D Fig). Glucose levels during GTT test in OB^[*Lep*]^ mice were statistically lower than OB^[3T3]^ mice (S4C Fig). Insulin tolerance tests (ITT) were not different between OB^Emp^, OB^[3T3]^, OB^[*Lep*]^ groups (Fig 5C and S4B, S4D–S4F Fig).

We also considered other key factors influencing glucose metabolism that are regulated by leptin, including insulin and glucagon [36]. Average insulin levels at sacrifice were 19.1 ± 5.7, 42.5 ± 28.9, 75.4 ± 43.9 ng/mL for OB^[Emp]^, OB^[3T3]^, and OB^[*Lep*]^ mice, respectively (Fig 5C). The insulin plasma concentration for OB^[*Lep*]^ was 4 times greater than OB^[Emp.^ However, the individual insulin concentrations varied considerably within groups and the trend for lower insulin in OB^[*Lep*]^ vs. OB^[Emp]^ was not statistically significant (Fig 5C). Glucagon levels of 1.11 ± 0.67, 0.86 ± 0.29, and 1.80 ± 0.87 ng/mL for OB^[Emp]^, OB^[3T3]^, and OB^[*Lep*]^, respectively were not statistically different (Fig 5E). We also examined resistin levels that is associated with increased insulin resistance in adipocytes [37]. Resistin is decreased by 30% in the presence of leptin (Fig 5E). Resistin concentration of 11.5 ± 3.1 ng/mL for OB^[*Lep*]^ was less than the 16.5 ± 3.2 and 16.9 ± 2.0 ng/mL for OB^[Emp]^ and OB^[3T3]^, respectively.

In previous studies [26], WT mice with a high-fat diet did not respond to treatment with leptin produced by gut cells. To test efficacy of leptin produced by [3T3^*Lep*^] adipocytes *in vivo*, we injected two groups of old WT mice with a high-fat diet-obesity and insulin resistance with encapsulated acellular capsules (WT^[Emp]^), and encapsulated 3T3^*Lep*^ (WT^[*Lep*]^) in both visceral fat pads (Fig 5F). WT^[*Lep*]^ mice had statistically lower AUC for glucose tolerance tests that was 30% less in WT^[*Lep*]^ than in WT^[Emp]^ (Fig 5F). Insulin tolerance tests were similar among all studied groups (Fig 5G). Thus, constitutive leptin production in encapsulated adipocytes was similarly efficient in improvement of glucose tolerance in mice with genetic or diet-induced obesity and insulin-resistance.

## Discussion

Two decades of research have established leptin as a key adipokine that regulates energy balance through the central nervous system and endocrine pathways [4, 36]. The leptin stimulated effects on the hypothalamus include: the regulation of appetite, an increase in metabolic rate, an increase in lipolysis within WAT, and thermogenesis [4]. These effects of leptin may have potential to be developed into a therapy for obesity. Recently, studies have shown that the hypothalamic action of leptin on BAT and muscles can mediate lifesaving and antidiabetic actions on both insulin-resistant and insulin-deficient rodents [38]. These studies provide a rationale for the translation of leptin into a complementary therapy to insulin treatments for type 1 and type 2 diabetes, which could improve the treatment of these diseases [36]. However, the therapeutic application of leptin is limited by the detrimental effects of daily leptin injections [27]. Injection site reactions often include inflammation, erythema and ecchymoses [27]. Furthermore, the systemic spike of plasma leptin injections can lead to hypoglycemia, and other complications [27]. Notably, hyperleptinemia has multiple deleterious effects [39, 40], including epigenetic and transgenerational effects, that promote obesity in offspring [41]. In 2014, leptin injections were approved by the United States and other countries for the treatment of congenital leptin deficiency and generalized lipodystrophy; however, the current methods of leptin delivery impede its use for the general population of patients with diabetes [41]. Physiologically, leptin is predominantly produced in circadian fashion in WAT [42]. In our study, we mimic physiological leptin production by employing encapsulated adipocytes producing leptin and by engrafting the capsules into a physiological location, the visceral fat. We showed that a sub-physiological concentration of leptin produced by these engrafts can effectively improve glucose tolerance in mice with genetic and diet-induced obesity.

The functions of leptin were established in *ob/ob* mice with systemic leptin deficiency and validated with the systemic injections of leptin or adenovirus producing leptin [25, 26, 28, 29]. The majority of systemic leptin effects (weight loss, thermogenesis, appetite suppression, and improved glucose metabolism) were recapitulated by the direct stimulation of the hypothalamus with leptin. Implants of intestinal cells overexpressing leptin showed effective appetite and weight loss in *ob/ob* mice [26]. Some insight into the tissues-specific effects of leptin was gained from these implantation studies. However, these implants failed to induce the same effects in WT mice with high-fat diet-induced obesity and insulin-resistance [26]. However, the data from this study need to be interpreted with caution because leptin production was induced with a drug. Moreover, genetically different implants in this study were susceptible to rejection by the host’s immune system, although they were introduced bound within an alginate polymer [26]. Other leptin replacement therapies have attempted to investigate endogenous leptin production by the transplantation of tissue [26, 30, 31]. However, these methods also induce an immune response. Furthermore, the transplantation of tissues producing leptin is unlikely to be a viable therapy for obese patients due to the health risks associated with transplantations. Encapsulation of cells, using alginate-poly-L-lysine, may overcome the immune response. The small size of the pores in the poly-L-lysine membrane excludes immunoglobulin influx [33, 43]. Encapsulated xenotransplants of porcine beta islets were able to survive and function in a human patient for over 9.5 years without immune rejection [43]. Research suggests that *ob/ob* mice have a weakened immune system [44, 45]. A weakened immune system may have allowed encapsulated cells to survive in *ob/ob* mice, but not in normal healthy WT mice. However, in a previous study in C57BL/6 mice encapsulated cells were able to avoid immune rejection as shown by foreign green fluorescent protein expression from the encapsulated cells weeks after injection [33]. Nevertheless, the effect that poly-L-lysine encapsulation has on the immune system and feasibility of using this encapsulation procedure should be fully elucidated in the future because of the possibility that the capsules survived due to a weaker immune system in *ob/ob* mice.

Encapsulation of adipocytes in our study, allowed the effects of constitutively produced leptin, within the visceral location, to be studied. In this pilot study, we introduced only 0.6 million cells per visceral depot and achieved only 10% of level of leptin observed in obese WT mice. Under these conditions, a limited leptin secretion from the capsules was not sufficient to reduce appetite and weight loss for the duration of the study. However, it had a profound effect on glucose metabolism, due to its systemic effects and effects in adipose tissue. This result also demonstrates that encapsulated adipocytes were able to evade the immune system, survive, and produce leptin for at least 72 days.

We attributed the transiently reduced weight and lower food intake, when compared to empty capsule injected control, to a combination of factors. The first factor was a response to the injections, since a decrease in food intake and weight was seen in all groups of mice during the first couple days post treatment. The second factor was related to implanted 3T3-L1 adipocytes. Food intake and weight of both OB^[*Lep*]^ and OB^[3T3]^ were significantly less than the empty (acellular) capsule injected group on various days. This transient weight loss and food intake reduction could be attributed to leptin as well as to the implantation of 3T3-L1 cells, because similar effects were reported after cross-transplantation of adipose tissue from different depots in *ob/ob* mice [30]. Furthermore, it is possible that 3T3-L1 adipocytes could secrete some leptin [46, 47]. Once 3T3-L1 adipocytes were implanted in the physiological VF environment, paracrine signaling to the encapsulated 3T3-L1 adipocytes could have been sufficient to release minimal amounts of leptin. The lack of the continual suppression of appetite and weight gain during our study could be due to the sub-physiologic leptin concentrations in plasma from small amount of encapsulated cells. It is also possible that leptin produced in the VF stimulates afferent neural responses that are not involved in the regulation of appetite and weight, because neural effects on weight loss seem to be uncoupled from other leptin-dependent effects [48].

The primary outcome of leptin production in encapsulated adipocytes in our study was the long-lasting improvement of glucose tolerance. This improvement was achieved by low, sub-physiological circulating levels of leptin. Three different mechanisms were at work and could be accounted for improved glucose uptake and utilization. We found: 1) increased BAT mass; 2) increased insulin; and 3) reduced resistin concentrations in plasma. Although WAT mass and liver fat accumulation was not influenced by leptin in *ob/ob* mice in our study, BAT mass was markedly greater in OB^[*Lep*]^ mice and associated with a higher basal metabolic rate. The greater RER in OB^[*Lep*]^ mice suggested the better glucose utilization that was demonstrated using GTT test. Recent studies showed that activation of γ-aminobutyric (GABA) and pro-opiomelanocorticitropin (POMC) neurons by leptin can induce BAT formation and function to increase glucose utilization [4]. The stimulation of innervation can potentially lead to improved secretion of insulin from pancreas, because studies in leptin-deficient β-cells suggest only a moderate direct effect of leptin in these cells [49]. Insulin secretion increases after long-term leptin administration [25]. Thus, both sub-physiological concentration of leptin from physiological location and systematic injections of high leptin concentrations may be effective in improving of insulin secretion.

Our study revealed also a new endocrine effect of leptin produced in VF location. OB^[*Lep*]^ mice secreted significantly less resistin compared to other treated groups. Resistin is an adipokine that was identified in visceral adipocytes as a key cytokine inducing insulin resistance [37]. Resistin deficiency in *ob*/*ob* mice and DIO mice have been shown to improve glucose tolerance [50]. Resistin is widely implicated in the development of and progression of insulin resistance in rodents and humans [37]. An antagonism between resistin and leptin secretion from adipocytes has been previously demonstrated *in vitro* [51]. Here we provide evidence that secretion of leptin from the small subset of encapsulated adipocytes is sufficient to mediate a long term decrease in resistin concentrations in plasma. The reduction of resistin has more beneficial outcome than the complete removal of resistin, because the complete removal of resistin decreases metabolic rate [50].

Our study shows that encapsulated adipocytes can produce leptin over 72 days. Although the amount of capsules could be optimized, we showed that sub-physiological levels of leptin produce an array of beneficial effects, notably long-term improved glucose tolerance. Leptin treatment is considered to be ineffective in the general population of patients with obesity and/or type 2 diabetes due to development of leptin resistance, hyperleptinemia, and insulin resistance [52]. This effect is also present in WT mice with diet-induced obesity (DIO) [52]. Previous strategies to improve glucose tolerance in DIO mice were ineffective [26]. However, more research is still needed to maximize the appetite suppressing and weight loss effects of encapsulated cells. This may include the further engineering of adipocytes to produce greater amounts of leptin or to develop other encapsulated leptin-producing cells, e.g. intestinal cells. In our study leptin, produced by encapsulated adipocytes in physiological location, led to long lasting improvement in glucose tolerance. Notably, this effect was achieved in severely obese old mice. More research is needed to establish the therapeutic effects and biocompatibility of encapsulated adipocytes to suggest that this delivery method offers advantages over standard delivery methods including injections, adenovirus delivery, or transplantation of tissue. However, encapsulation is a minimally invasive compared to surgical interventions and may be a cost-effective procedure for delivery of leptin at a constant rate over a prolonged period of time from months [33] to years [43] as well as to the termination of this procedure [33]. Our data provide a proof of principle that the encapsulation of leptin-producing adipocytes could be developed. Encapsulation is a feasible translational approach for the controlled delivery of genes encoding adipokines into adipose tissue.

## Materials and Methods

### Chemicals and reagents

Unless otherwise noted, we purchased chemicals and reagents from Sigma-Aldrich (St. Louis, MO) and cell culture media from Invitrogen (Carlsbad, CA).

### Cell line engineering

To derive a leptin overexpressing 3T3-L1 preadipocyte cell line, we performed a stable transfection of 3T3-L1 (ATCC CL-173, obtained 8/2014 from the American Type Culture Collection, Manassas, VA) using an untagged overexpressing leptin clone (GeneCopoeia, Rockville, MD, catalog number EX-Mm03615-Lv67, CMV promoter, puromycin resistant) packaged in a Lenti-Pac FIV Expression Packaging Kit (GeneCopoeia, Rockville, MD) according to manufacturer’s instructions. Briefly, lentiviral titers were produced using Lenti-Pac^™^ Lentiviral Packaging Kits (GeneCopoeia, Rockville, MD). A total of 1.3–1.5 × 10^6^ of the HEK293Ta Lentiviral Packaging cells (GeneCopoeia, Rockville, MD) were plated in a 10-cm dish 2 days before transfection in DMEM (10 mL), which was supplemented with 10% heat-inactivated fetal bovine serum (FBS). Puromycin resistant lentiviral ORF leptin cDNA clone (2.5 μg; GeneCopoeia, Rockville, MD), 5 μL Lenti-Pac FIV mix, and 15 μL of Endofectin–Lenti were diluted into 200 μL Opti-MEMI (Invitrogen, Grand Island, NY). The DNA–EndoFectin–Lenti complex was incubated at room temperature for 15 min and then added directly to the cells. Cells were incubated in a CO_2_ incubator at 37°C for 8–14 h. TiterBoost reagent (GeneCopoeia, Rockville, MD, 0.2%) was added to the culture medium. Culture medium containing leptin pseudovirus vector was collected 48 h post transfection after being centrifuged at 2000 rpm for 15 min. and used for the transfection. Murine 3T3-L1 preadipocytes were grown in a 6-well plate until 80% confluent. Then they were transfected with 1 mL of the vector-containing supernatant, 0.5 μL Polybrene (Millipore, Billerica, MA) in 0.5mL 10% calf serum (CS). 24 h post transfection, cells were replaced with a standard culture medium (DMEM containing 10% CS and 1% penicillin-streptomycin). At 90% confluence, cells were plated into 96-well plate with puromycin (1.0 mg/mL, Invitrogen, Grand Island, NY). The survived cells were plated in 96 well to achieve a density of single cell per well. Stably transfected overexpressing leptin clones (3T3^*Lep*^) were derived from a single cell and were tested for leptin mRNA expression by RT-PCR and leptin release by ELISA (ALPCO, Salem, NH).

### mRNA analysis

mRNA was purified from adipocytes or adipose tissue according to the manufacturer’s instructions (Qiagen, Germantown, MD) and quantified using 7900HT Fast Real-Time PCR System and TaqMan fluorogenic detection system (Applied Biosystems, Grand Island, NY). The *Lep* (Assay ID: Mm00434759_m1), *Pref1* (Assay ID: Mm00494477_m1), and *Pparg* (Assay ID: Mm00440945_m2) validated primers were also purchased from Applied Biosystems (Grand Island, NY). Comparative real-time PCR was performed in triplicate, including no-template controls. Expression was calculated using the comparative Ct method normalized to the TATA box binding protein (TBP, Applied Biosystems, Grand Island, NY, Assay ID: Mm00446973_m1).

### Cell differentiation

All preadipocytes (3T3^*Lep*^ and parent 3T3-L1 cells) were cultured in DMEM medium containing 10% calf serum. Lipogenic differentiation medium contained 10% FBS, 10μg/mL insulin, 1μM dexamethasone, 0.5mM 3-isobutyl-1-methyl xanthine. Medium was replaced every 48 hours with DMEM containing 10% FBS, 10μg/mL insulin, and continued for 7 days.

### Encapsulation

The phase microencapsulation technique has been performed as described [53]. The cell suspension (1 x 10^6^ cells/ml in 2% sodium alginate solution) was extruded through a 0.4-mm needle into a 100mM CaCl_2_ solution, using Encapsulation Unit (Nisco Engineering, AG, Switzerland) at 5.4kV to form calcium alginate gel beads. Microbeads were solidified for 20 min, and then incubated with 0.05% (w/v) poly-L-lysine (MW 20,700) to form alginate-poly-L-lysine membrane around the surface. The core was liquefied using 50mM sodium citrate. Capsules′ properties were characterized before [53]. Encapsulated preadipocytes were maintained in culture under standard conditions for up to 30d.

### Animal studies

All experimental protocols were approved by the Institutional Animal Care and Use Committee at the Ohio State University.

#### Mouse study one

Five week old *ob/ob* male mice (B6.Cg-Lep<ob>/J, The Jackson Laboratory, Bar Harbor, ME, n = 21) were purchased, housed individually, and randomly assigned into three groups and fed *ad libitum* a standard chow diet (irradiated 7912, Harlan Laboratories, Indianapolis, IL) for 72 days. The following treatment groups were studied:

1. Control treatment group (OB^[Emp]^) (n = 5 at the conclusion of the study). Male *ob/ob* mice were injected with vehicle (0.3 mL of acellular (empty) capsules in 0.5 mL of sterile phosphate buffer (PBS) per visceral depot).
2. Encapsulated 3T3-L1 group (OB^[3T3]^) (n = 5 at the conclusion of the study). Male *ob/ob* mice were injected with encapsulated 3T3-L1 preadipocytes (0.3*10^6^ 3T3-L1 cells in 0.3 mL of capsules in 0.5 mL of PBS per visceral depot).
3. Encapsulated 3T3^*Lep*^ group. (OB^[*Lep*]^) (n = 7 at the conclusion of the study). Male *ob/ob* mice were injected with encapsulated 3T3^*Lep*^ preadipocytes overexpressing leptin (0.3*10^6^ 3T3^*Lep*^ cells in 0.3mL of capsules in 0.5 mL of PBS per visceral depot).

All mice assigned to OB^[Emp]^, OB^[3T3]^, and OB^[*Lep*]^ groups had similar weights before treatments of 56.3, 56.8, and 58.0 g, respectively(*P*<0.80, ANOVA). After injection, mice continued on a similar chow diet for 72 days and then sacrificed. Food intake and weight was monitored. On day 28, mice were removed from standard housing and placed in metabolic cages for two days, then returned to individual housing. Glucose tolerance tests (GTT) and insulin tolerance tests (ITT) were performed three weeks prior to sacrifice. DEXA was performed on OB^[Emp]^, OB^[3T3]^, and OB^[*Lep*]^ just prior to sacrifice using the GE Lunar Prodigy (Fairfield, CT). Percent body fat was measured using enCORE software (Fairfield, CT). On day 72, mice were anesthetized using isoflurane (Piramal Healthcare Limited, Andhra Pradesh, India) and sacrificed. Death was confirmed by removal of vital organs. Blood was collected by cardiac puncture into EDTA-containing tubes. Plasma was prepared by centrifugation. One whole subcutaneous (inguinal, lower back) and visceral fat (perigonadal/epididymal) pads were dissected as shown in [35] and homogenized in RIPA buffer with protease inhibitors.

#### Mouse study two

Eight C57BL/6 (WT) five week old male mice (The Jackson Laboratory, Bar Harbor, ME) were fed a high-fat (HF) diet (45% kcal from fat, D12451, Research Diets Inc., New Brunswick, NJ) for 218 days. Mice were housed together in two groups and food intake and weight was recorded. Treatments were:

1. Control treatment group (WT^Emp^) (n = 3). Body weight at the beginning of study was 56.3±2.9g. WT male mice were injected with vehicle (0.3 mL of acellular (empty) capsules in 0.5 mL sterile phosphate buffer (PBS) per visceral depot);
2. [3T3^*Lep*^] treatment group (WT^[*Lep*]^) (n = 5). Body weight at the beginning of study was 58.0±3.3g WT male mice were injected with encapsulated 3T3^*Lep*^ preadipocytes overexpressing leptin (0.3*10^6^ 3T3^*Lep*^ cells in 0.3 mL of capsules in 0.5 mL of PBS per visceral depot).

After injection, mice continued on the same HF chow diet for 70 days and glucose and insulin tolerance tests were performed. Prior to sacrifice, the three heaviest mice in the WT^[*Lep*]^ group died before the end of study and tissues were unable to be harvested prior to degradation.

### Glucose and insulin tolerance tests

For GTT and ITT, mice were fasted overnight. They were injected with a single intraperitoneal glucose dose (0.001g glucose/g body weight) for GTT. Blood was obtained from mouse tails. Blood glucose was measured by One Touch Ultra glucometer (LifeScan). For mice were fasted overnight. Mice allowed recovered for at least one week, then ITT test was performed using a single intraperitoneal insulin dose (1mU of insulin/g body weight). Blood glucose was measured.

### Western blot analysis

Aliquots from fat pad homogenates were used for protein (Bicinchoninic Acid Kit, Thermo Scientific, Waltham, MA) measurements, and Western blots. For Western blot analyses, tissue lysates were separated on 10% acrylamide gel under reducing conditions. After transfer to polyvinylidene fluoride membranes (Immobilon-P, Millipore, Billerica, MA), the membranes were stained with primary antibodies against UCP1 (Abcam, Cambridge, MA), PGC-1α (Abcam, Cambridge, MA), and β-actin (Cell Signaling, Danvers, MA) and then detected with the appropriate secondary antibody (LI-COR, Lincoln, NE). Proteins were analyzed using an Odyssey Infrared Imaging System (LI-COR, Lincoln, NE). Images were quantified by ImageJ software.

### Metabolic measurements

In study one, metabolic parameters in the treated mice were measured by indirect calorimetry (CLAMS, Columbus Instruments, Columbus, OH) at ambient temperature (22°C) with 12 hours light/dark cycles. Animals were fed the same diet and water provided *ad libitum*. Mice were placed individually and allowed to acclimate to the metabolic cages. O_2_ consumption, CO_2_ production, energy expenditure, and locomotor activity were measured for 24 hours. Based on these data, respiratory quotient or exchange ratio (CO_2_/O_2_) and Δ heat values were calculated by CLAMS.

### ELISA assays

Leptin (mouse/rat) ELISA (ALPCO, Salem, NH) was performed on media samples and plasma samples. 3T3-L1 and 3T3^*Lep*^ were differentiated as described (n = 5). 2 mL of fresh media was added to the differentiated cells and incubated for 48 h. Media samples were collected and leptin (mouse/rat) ELISA (ALPCO, Salem, NH) was performed according to manufacturer’s instructions. 3T3-L1 and 3T3^*Lep*^ were encapsulated in poly-L-lysine as described. 200μL of encapsulated 3T3-L1 ([3T3-L1]) or encapsulated 3T3^*Lep*^ ([3T3^*Lep*^]) were added to 2mL of fresh cell culture medium (DMEM medium containing 10% CS). After 24 h incubation, media samples were taken, centrifuged, and filtered to remove capsules. Leptin (mouse/rat) concentrations in the media were measured by ELISA (ALPCO, Salem, NH) according to the manufacturer’s instruction. Insulin concentration in plasma (diluted 1 to 20) was determined using the Rat/Mouse Insulin ELISA Kit (EMD Millipore Corporation, St. Charles, MO). Resistin concentration in plasma (diluted 1 to 50) was determined using Quantikine ELISA Mouse Resistin Immunoassay (R&D Systems, INC., Minneapolis, MN). Hemolyzed plasma samples were excluded from measurements according to manufacturers’ recommendations.

IL-6 concentration in plasma (diluted 1 to 5) and in VF (30μg of protein per sample) was determined using Quantikine ELISA Mouse IL-6 Immunoassay (R&D Systems, INC., Minneapolis, MN). Hemolyzed plasma samples were excluded from measurements according to manufacturers’ recommendations.

### Statistical analysis

Data are shown as mean±SD of experiments that were performed at least with n = 3 for clone characterization *in vitro* and n = 5 *in vivo* unless otherwise noted. Group comparisons of three or more independent groups were performed using one-way ANOVA followed by Tukey’s post hoc test unless otherwise indicated. For comparisons of two independent groups, the Student’s *t*-test was used unless otherwise indicated. Significance level was set at an alpha = 0.05 unless otherwise noted.

## Supporting Information

S1 Fig Kinetics of food consumption and weight, liver weight, and UCP1 expression in BAT. O*b/ob* mice (n = 7 per group) were injected with encapsulated acellular capsules (OB^[Emp]^), encapsulated 3T3-L1 preadipocytes (OB^[3T3]^), and encapsulated 3T3^*Lep*^ (OB^[*Lep*]^) in both visceral fat pads (total 0.6*10^6^ adipocytes for each cell type). (A) Average food intake kinetics in OB^[Emp]^ (open circles, n = 6) and OB^[*Lep*]^ (closed circles, n = 7) mice is shown as percent of food intake prior to injection in pre-and post-injection period. Arrow indicates the day of injection. Asterisks show *P<*0.05, Student’s t-test. (B) Average weight kinetics in same mice. Weight is shown as percent of weight prior to injection in pre-and post-injection period. (C) Average food intake kinetics in OB^[Emp]^ (open circles, n = 6) and OB^[3T3]^ (closed squares, n = 6). (D) Average weight kinetics in same mouse groups. (E) Average normalized weight of liver to body weight in same mouse groups. All data are represented as mean ± SD. Significant (*P<*0.05, Student’s t-test) statistical comparisons between groups are shown with an asterisk.
(TIF)

S2 Fig The lack of leptin effect on intraleukin-6 (Il-6) levels in plasma and UCP1 and PGC1α protein expression in brown adipose tissue. (A) IL-6 levels was measured in plasma by ELISA. (n = 5 per OB^[Emp]^ and OB^[*Lep*]^; n = 7 per OB^[3T3]^ group). (B-D) Representative western blot (B) and relative protein expression of PGC1α (C) and UCP1 (D) and in BAT in all treated groups (n = 5 per OB^[Emp]^ and OB^[3T3]^; n = 6 per OB^[*Lep*]^ group). Data (mean ± SD) show the ratio of PGC1α or UCP1 to β-actin.
(TIF)

S3 Fig Leptin increases metabolic rate and raises RER. (A-D) Metabolic measurements were performed in OB^[3T3]^ (white bar, n = 6) and OB^[*Lep*]^ (black bar, n = 6) mouse groups in CLAMS metabolic cages at 28 days post injection. The average (mean ± SD) x, y, and z activity (A), average VO_2_ (mL/kg/h) (B), respiratory exchange ratio (RER) (C), and average metabolic rate (kcal/h/kg) (D) are shown for both the dark (D) and light (L) cycles. (E, F) Kinetic data for RER (E) and metabolic rate (kcal/h/kg) (F) are shown as mean for each time point in OB^[3T3]^ (open black circles) and OB^[*Lep*]^ (filled red circles) groups. (E, F) Kinetic data for RER (E) and metabolic rate (kcal/h/kg) (F) are shown as mean for each time point in OB^[3T3]^ (open black circles) and OB^[*Lep*]^ (filled red circles) groups.
(TIF)

S4 Fig Leptin improves glucose tolerance. (A, B) Glucose tolerance tests (GTT) (A) or insulin tolerance tests (ITT) (B) were performed in fasting overnight mice OB^[3T3]^ (open circles, n = 5) and OB^[*Lep*]^ (filled circles, n = 5). Average blood glucose (mg/dL) are displayed for each time point. Asterisks indicate significant differences *P*<0.05, Student’s t-test. (C) The area under the curve (AUC) for each ITT was calculated using a trapezoidal approximation and displayed as percent of the control OB^[Emp]^ (n = 5) group (100%). (D). WT mice with diet-induced obesity (Study 2) were injected with encapsulated acellular capsules (WT^[Emp]^) and encapsulated 3T3^*Lep*^ (WT^[*Lep*]^) in both visceral fat pads (total 0.6*10^6^ adipocytes for each cell type). ITT were performed in fasting overnight mice. Average AUC (mean ± SD) are shown for WT^[Emp]^ (white bar, n = 3) and WT^[*Lep*]^ (black bar, n = 5).
(TIF)

## References

[1] Ogden CL, Carroll MD, Kit BK, Flegal KM. Prevalence of Obesity in the United States, 2009–2010. NCHS Data Brief. 2012;(82). 22617494

[2] Ginter E, Simko V. Type 2 diabetes mellitus, pandemic in 21st century. Advances in experimental medicine and biology. 2012;771:42–50. . 23393670 10.1007/978-1-4614-5441-0_6

[3] Yang W, Lu J, Weng J, Jia W, Ji L, Xiao J, et al Prevalence of diabetes among men and women in China. N Engl J Med. 2010;362(12):1090–101. 10.1056/NEJMoa0908292 . 20335585

[4] Fujikawa T, Berglund ED, Patel VR, Ramadori G, Vianna CR, Vong L, et al Leptin engages a hypothalamic neurocircuitry to permit survival in the absence of insulin. Cell Metab. 2013;18(3):431–44. 10.1016/j.cmet.2013.08.004 24011077 PMC3890693

[5] Orchard TJ, Olson JC, Erbey JR, Williams K, Forrest KY, Smithline Kinder L, et al Insulin resistance-related factors, but not glycemia, predict coronary artery disease in type 1 diabetes: 10-year follow-up data from the Pittsburgh Epidemiology of Diabetes Complications Study. Diabetes Care. 2003;26(5):1374–9. . 12716791 10.2337/diacare.26.5.1374

[6] Maffei M, Halaas J, Ravussin E, Pratley RE, Lee GH, Zhang Y, et al Leptin levels in human and rodent: measurement of plasma leptin and ob RNA in obese and weight-reduced subjects. Nature medicine. 1995;1(11):1155–61. Epub 1995/11/01. . 7584987 10.1038/nm1195-1155

[7] Maffei M, Fei H, Lee GH, Dani C, Leroy P, Zhang Y, et al Increased expression in adipocytes of ob RNA in mice with lesions of the hypothalamus and with mutations at the db locus. Proc Natl Acad Sci U S A. 1995;92(15):6957–60. Epub 1995/07/18. 7624352 10.1073/pnas.92.15.6957 PMC41450

[8] Stephens TW, Basinski M, Bristow PK, Bue-Valleskey JM, Burgett SG, Craft L, et al The role of neuropeptide Y in the antiobesity action of the obese gene product. Nature. 1995;377(6549):530–2. Epub 1995/10/12. 10.1038/377530a0 . 7566151

[9] Woods AJ, Stock MJ. Leptin activation in hypothalamus. Nature. 1996;381(6585):745 Epub 1996/06/27. 10.1038/381745a0 . 8657278

[10] Glaum SR, Hara M, Bindokas VP, Lee CC, Polonsky KS, Bell GI, et al Leptin, the obese gene product, rapidly modulates synaptic transmission in the hypothalamus. Molecular pharmacology. 1996;50(2):230–5. Epub 1996/08/01. . 8700128

[11] Costa A, Poma A, Martignoni E, Nappi G, Ur E, Grossman A. Stimulation of corticotrophin-releasing hormone release by the obese (ob) gene product, leptin, from hypothalamic explants. Neuroreport. 1997;8(5):1131–4. Epub 1997/03/24. . 9175099 10.1097/00001756-199703240-00014

[12] Haque MS, Minokoshi Y, Hamai M, Iwai M, Horiuchi M, Shimazu T. Role of the sympathetic nervous system and insulin in enhancing glucose uptake in peripheral tissues after intrahypothalamic injection of leptin in rats. Diabetes. 1999;48(9):1706–12. Epub 1999/09/10. . 10480598 10.2337/diabetes.48.9.1706

[13] Minokoshi Y, Haque MS, Shimazu T. Microinjection of leptin into the ventromedial hypothalamus increases glucose uptake in peripheral tissues in rats. Diabetes. 1999;48(2):287–91. Epub 1999/05/20. . 10334303 10.2337/diabetes.48.2.287

[14] Satoh N, Ogawa Y, Katsuura G, Numata Y, Tsuji T, Hayase M, et al Sympathetic activation of leptin via the ventromedial hypothalamus: leptin-induced increase in catecholamine secretion. Diabetes. 1999;48(9):1787–93. Epub 1999/09/10. . 10480609 10.2337/diabetes.48.9.1787

[15] Zeng W, Pirzgalska RM, Pereira MM, Kubasova N, Barateiro A, Seixas E, et al Sympathetic Neuro-adipose Connections Mediate Leptin-Driven Lipolysis. Cell. 2015;163(1):84–94. Epub 2015/09/26. 10.1016/j.cell.2015.08.055 . 26406372 PMC7617198

[16] Dodd GT, Decherf S, Loh K, Simonds SE, Wiede F, Balland E, et al Leptin and insulin act on POMC neurons to promote the browning of white fat. Cell. 2015;160(1–2):88–104. Epub 2015/01/17. 10.1016/j.cell.2014.12.022 25594176 PMC4453004

[17] Farooqi IS, Matarese G, Lord GM, Keogh JM, Lawrence E, Agwu C, et al Beneficial effects of leptin on obesity, T cell hyporesponsiveness, and neuroendocrine/metabolic dysfunction of human congenital leptin deficiency. J Clin Invest. 2002;110(8):1093–103. Epub 2002/10/24. 10.1172/jci15693 12393845 PMC150795

[18] Gibson WT, Farooqi IS, Moreau M, DePaoli AM, Lawrence E, O'Rahilly S, et al Congenital leptin deficiency due to homozygosity for the Delta133G mutation: report of another case and evaluation of response to four years of leptin therapy. J Clin Endocrinol Metab. 2004;89(10):4821–6. Epub 2004/10/09. 10.1210/jc.2004-0376 . 15472169

[19] Oral EA, Simha V, Ruiz E, Andewelt A, Premkumar A, Snell P, et al Leptin-replacement therapy for lipodystrophy. N Engl J Med. 2002;346(8):570–8. Epub 2002/02/22. 10.1056/NEJMoa012437 . 11856796

[20] Ingalls AM, Dickie MM, Snell GD. Obese, a new mutation in the house mouse. The Journal of heredity. 1950;41(12):317–8. Epub 1950/12/01. . 14824537 10.1093/oxfordjournals.jhered.a106073

[21] Batchelor BR, Stern JS, Johnson PR, Mahler RJ. Effects of streptozotocin on glucose metabolism, insulin response, and adiposity in ob/ob mice. Metabolism. 1975;24(1):77–91. Epub 1975/01/01. . 122852 10.1016/0026-0495(75)90009-8

[22] Surwit RS, McCubbin JA, Livingston EG, Feinglos MN. Classically conditioned hyperglycemia in the obese mouse. Psychosomatic medicine. 1985;47(6):565–8. Epub 1985/11/01. . 4070525 10.1097/00006842-198511000-00006

[23] Mistry AM, Swick AG, Romsos DR. Leptin rapidly lowers food intake and elevates metabolic rates in lean and ob/ob mice. The Journal of nutrition. 1997;127(10):2065–72. Epub 1997/10/06. . 9311966 10.1093/jn/127.10.2065

[24] Harris RB, Zhou J, Redmann SM Jr., Smagin GN, Smith SR, Rodgers E, et al A leptin dose-response study in obese (ob/ob) and lean (+/?) mice. Endocrinology. 1998;139(1):8–19. . 9421392 10.1210/endo.139.1.5675

[25] Khan A, Narangoda S, Ahren B, Holm C, Sundler F, Efendic S. Long-term leptin treatment of ob/ob mice improves glucose-induced insulin secretion. Int J Obes Relat Metab Disord. 2001;25(6):816–21. Epub 2001/07/06. 10.1038/sj.ijo.0801628 . 11439295

[26] Oosman SN, Lam AW, Harb G, Unniappan S, Lam NT, Webber T, et al Treatment of obesity and diabetes in mice by transplant of gut cells engineered to produce leptin. Mol Ther. 2008;16(6):1138–45. Epub 2008/04/17. mt200862 [pii] 10.1038/mt.2008.62 . 18414479

[27] Baldo BA. Side effects of cytokines approved for therapy. Drug safety. 2014;37(11):921–43. 10.1007/s40264-014-0226-z . 25270293 PMC7101846

[28] Morsy MA, Gu MC, Zhao JZ, Holder DJ, Rogers IT, Pouch WJ, et al Leptin gene therapy and daily protein administration: a comparative study in the ob/ob mouse. Gene therapy. 1998;5(1):8–18. Epub 1998/04/16. 10.1038/sj.gt.3300565 . 9536260

[29] Muzzin P, Eisensmith RC, Copeland KC, Woo SL. Correction of obesity and diabetes in genetically obese mice by leptin gene therapy. Proc Natl Acad Sci U S A. 1996;93(25):14804–8. Epub 1996/12/10. 8962136 10.1073/pnas.93.25.14804 PMC26217

[30] Klebanov S, Astle CM, DeSimone O, Ablamunits V, Harrison DE. Adipose tissue transplantation protects ob/ob mice from obesity, normalizes insulin sensitivity and restores fertility. J Endocrinol. 2005;186(1):203–11. Epub 2005/07/09. 10.1677/joe.1.06150 . 16002549

[31] Liu X, Wang S, You Y, Meng M, Zheng Z, Dong M, et al Brown Adipose Tissue Transplantation Reverses Obesity in Ob/Ob Mice. Endocrinology. 2015;156(7):2461–9. Epub 2015/04/02. 10.1210/en.2014-1598 . 25830704

[32] Xu L, Shen Q, Mao Z, Lee LJ, Ziouzenkova O. Encapsulation Thermogenic Preadipocytes for Transplantation into Adipose Tissue Depots. Journal of visualized experiments: JoVE. 2015;(100):e52806 10.3791/52806 26066392 PMC4467455

[33] Yang F, Zhang X, Maiseyeu A, Mihai G, Yasmeen R, DiSilvestro D, et al The prolonged survival of fibroblasts with forced lipid catabolism in visceral fat following encapsulation in alginate-poly-L-lysine. Biomaterials. 2012;33(22):5638–49. Epub 2012/05/12. 10.1016/j.biomaterials.2012.04.035 . 22575837 PMC3815596

[34] Wang Y, Kim KA, Kim JH, Sul HS. Pref-1, a preadipocyte secreted factor that inhibits adipogenesis. The Journal of nutrition. 2006;136(12):2953–6. Epub 2006/11/23. . 17116701 10.1093/jn/136.12.2953

[35] Yasmeen R, Reichert B, Deiuliis J, Yang F, Lynch A, Meyers J, et al Autocrine Function of Aldehyde Dehydrogenase 1 as a Determinant of Diet- and Sex-Specific Differences in Visceral Adiposity. Diabetes. 2012 Epub 2012/08/31. 10.2337/db11-1779 . 22933113 PMC3526050

[36] Unger RH, Roth MG. A new biology of diabetes revealed by leptin. Cell Metab. 2015;21(1):15–20. 10.1016/j.cmet.2014.10.011 . 25456738

[37] Steppan CM, Bailey ST, Bhat S, Brown EJ, Banerjee RR, Wright CM, et al The hormone resistin links obesity to diabetes. Nature. 2001;409(6818):307–12. Epub 2001/02/24. 10.1038/35053000 . 11201732

[38] Yu X, Park BH, Wang MY, Wang ZV, Unger RH. Making insulin-deficient type 1 diabetic rodents thrive without insulin. Proc Natl Acad Sci U S A. 2008;105(37):14070–5. 10.1073/pnas.0806993105 18779578 PMC2544580

[39] Gonzalez M, Lind L, Soderberg S. Leptin and endothelial function in the elderly: the Prospective Investigation of the Vasculature in Uppsala Seniors (PIVUS) study. Atherosclerosis. 2013;228(2):485–90. 10.1016/j.atherosclerosis.2013.03.018 . 23591414

[40] Kshatriya S, Reams GP, Spear RM, Freeman RH, Dietz JR, Villarreal D. Obesity hypertension: the emerging role of leptin in renal and cardiovascular dyshomeostasis. Curr Opin Nephrol Hypertens. 2010;19(1):72–8. 10.1097/MNH.0b013e328332fb49 . 19851106

[41] Pollock KE, Stevens D, Pennington KA, Thaisrivongs R, Kaiser J, Ellersieck MR, et al Hyperleptinemia During Pregnancy Decreases Adult Weight of Offspring and Is Associated With Increased Offspring Locomotor Activity in Mice. Endocrinology. 2015;156(10):3777–90. 10.1210/en.2015-1247 . 26196541

[42] Herrero L, Valcarcel L, da Silva CA, Albert N, Diez-Noguera A, Cambras T, et al Altered circadian rhythm and metabolic gene profile in rats subjected to advanced light phase shifts. PloS one. 2015;10(4):e0122570 10.1371/journal.pone.0122570 25837425 PMC4383616

[43] Elliott RB, Escobar L, Tan PL, Muzina M, Zwain S, Buchanan C. Live encapsulated porcine islets from a type 1 diabetic patient 9.5 yr after xenotransplantation. Xenotransplantation. 2007;14(2):157–61. Epub 2007/03/27. XEN384 [pii] 10.1111/j.1399-3089.2007.00384.x . 17381690

[44] Bernotiene E, Palmer G, Gabay C. The role of leptin in innate and adaptive immune responses. Arthritis research & therapy. 2006;8(5):217 Epub 2006/08/02. 10.1186/ar2004 16879738 PMC1779438

[45] Fantuzzi G, Faggioni R. Leptin in the regulation of immunity, inflammation, and hematopoiesis. Journal of leukocyte biology. 2000;68(4):437–46. Epub 2000/10/19. . 11037963

[46] Norman D, Isidori AM, Frajese V, Caprio M, Chew SL, Grossman AB, et al ACTH and alpha-MSH inhibit leptin expression and secretion in 3T3-L1 adipocytes: model for a central-peripheral melanocortin-leptin pathway. Molecular and cellular endocrinology. 2003;200(1–2):99–109. Epub 2003/03/20. . 12644303 10.1016/s0303-7207(02)00410-0

[47] Zeigerer A, Rodeheffer MS, McGraw TE, Friedman JM. Insulin regulates leptin secretion from 3T3-L1 adipocytes by a PI 3 kinase independent mechanism. Exp Cell Res. 2008;314(11–12):2249–56. Epub 2008/05/27. 10.1016/j.yexcr.2008.04.003 18501893 PMC2997521

[48] Turner RT, Dube M, Branscum AJ, Wong CP, Olson DA, Zhong X, et al Hypothalamic leptin gene therapy reduces body weight without accelerating age-related bone loss. J Endocrinol. 2015;227(3):129–41. 10.1530/JOE-15-0280 . 26487675 PMC4917201

[49] Soedling H, Hodson DJ, Adrianssens AE, Gribble FM, Reimann F, Trapp S, et al Limited impact on glucose homeostasis of leptin receptor deletion from insulin- or proglucagon-expressing cells. Mol Metab. 2015;4(9):619–30. 10.1016/j.molmet.2015.06.007 26413468 PMC4563029

[50] Qi Y, Nie Z, Lee YS, Singhal NS, Scherer PE, Lazar MA, et al Loss of resistin improves glucose homeostasis in leptin deficiency. Diabetes. 2006;55(11):3083–90. Epub 2006/10/27. 10.2337/db05-0615 . 17065346

[51] Ye F, Than A, Zhao Y, Goh KH, Chen P. Vesicular storage, vesicle trafficking, and secretion of leptin and resistin: the similarities, differences, and interplays. J Endocrinol. 2010;206(1):27–36. 10.1677/JOE-10-0090 . 20392813

[52] Bence KK, Delibegovic M, Xue B, Gorgun CZ, Hotamisligil GS, Neel BG, et al Neuronal PTP1B regulates body weight, adiposity and leptin action. Nature medicine. 2006;12(8):917–24. 10.1038/nm1435 . 16845389

[53] Zhang X, He H, Yen C, Ho W, Lee LJ. A biodegradable, immunoprotective, dual nanoporous capsule for cell-based therapies. Biomaterials. 2008;29(31):4253–9. Epub 2008/08/13. S0142-9612(08)00511-5 [pii] 10.1016/j.biomaterials.2008.07.032 . 18694595

