# Supplementary figures and images for: Leptin Production by Encapsulated Adipocytes Increases Brown Fat, Decreases Resistin, and Improves Glucose Intolerance in Obese Mice
Source: PLoS One. 2016 Apr 7;11(4):e0153198. doi: 10.1371/journal.pone.0153198 (PMC4824514; doi:10.1371/journal.pone.0153198)

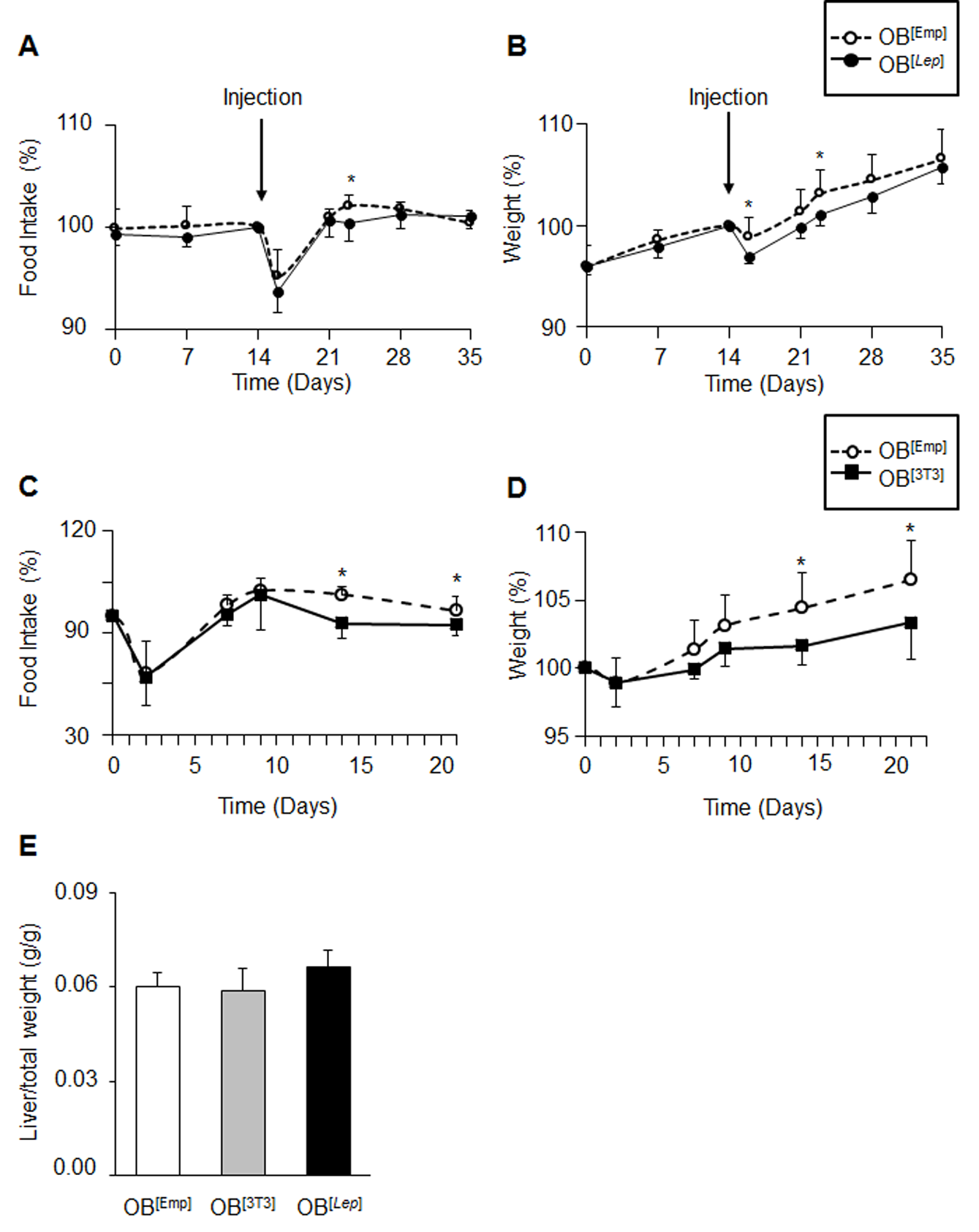

Supplement: S1 Fig — Ob/ob mice (n = 7 per group) were injected with encapsulated acellular capsules (OB[Emp]), encapsulated 3T3-L1 preadipocytes (OB[3T3]), and encapsulated 3T3Lep (OB[Lep]) in both visceral fat pads (total 0.6*106 adipocytes for each cell type). (A) Average food intake kinetics in OB[Emp] (open circles, n = 6) and OB[Lep] (closed circles, n = 7) mice is shown as percent of food intake prior to injection in pre-and post-injection period. Arrow indicates the day of injection. Asterisks show P<0.05, Student’s t-test. (B) Average weight kinetics in same mice. Weight is shown as percent of weight prior to injection in pre-and post-injection period. (C) Average food intake kinetics in OB[Emp] (open circles, n = 6) and OB[3T3] (closed squares, n = 6). (D) Average weight kinetics in same mouse groups. (E) Average normalized weight of liver to body weight in same mouse groups. All data are represented as mean ± SD. Significant (P<0.05, Student’s t-test) statistical comparisons between groups are shown with an asterisk. (TIF) [file pone.0153198.s001.tif]

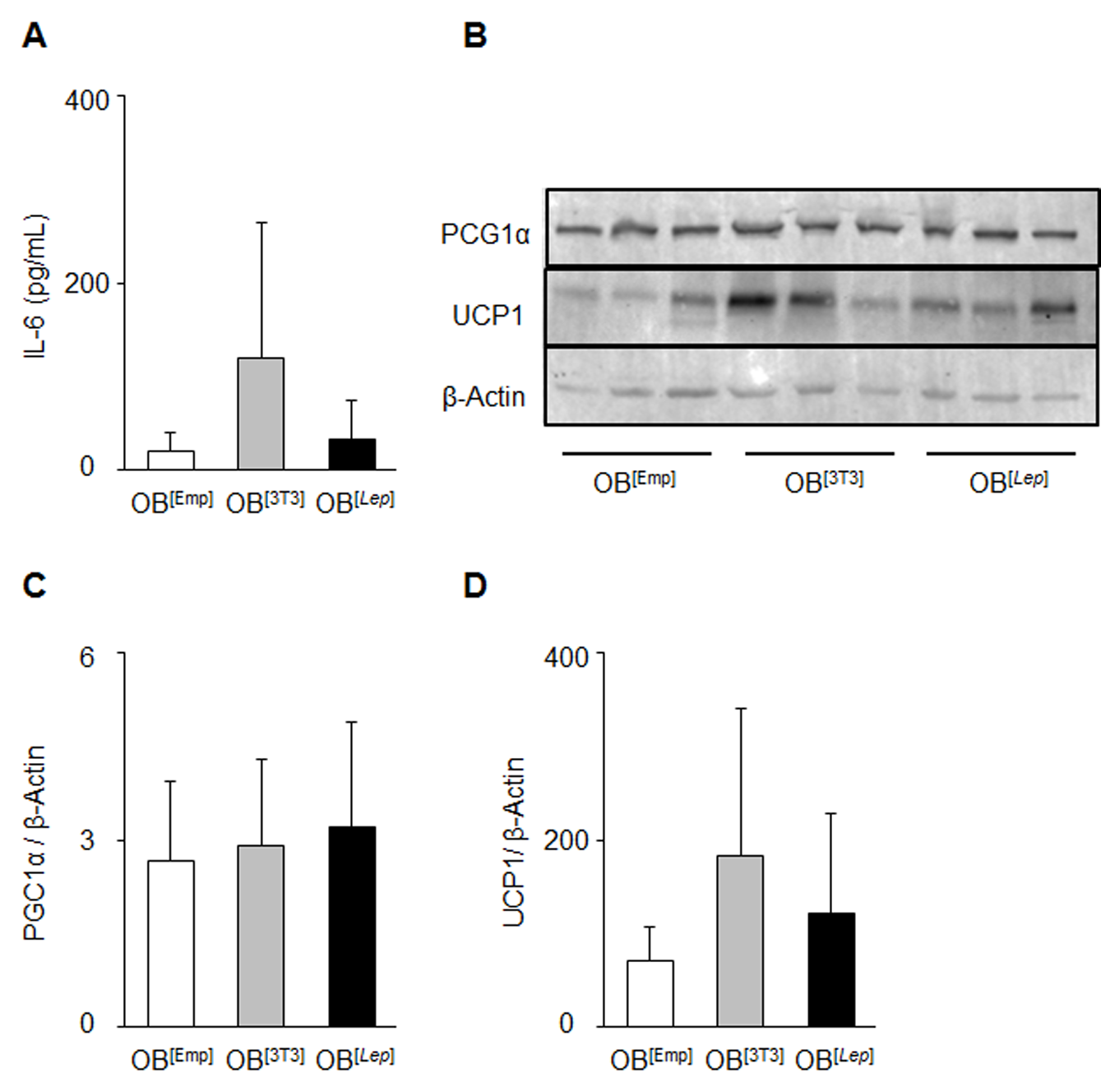

Supplement: S2 Fig — (A) IL-6 levels was measured in plasma by ELISA. (n = 5 per OB[Emp] and OB[Lep]; n = 7 per OB[3T3] group). (B-D) Representative western blot (B) and relative protein expression of PGC1α (C) and UCP1 (D) and in BAT in all treated groups (n = 5 per OB[Emp] and OB[3T3]; n = 6 per OB[Lep] group). Data (mean ± SD) show the ratio of PGC1α or UCP1 to β-actin. (TIF) [file pone.0153198.s002.tif]

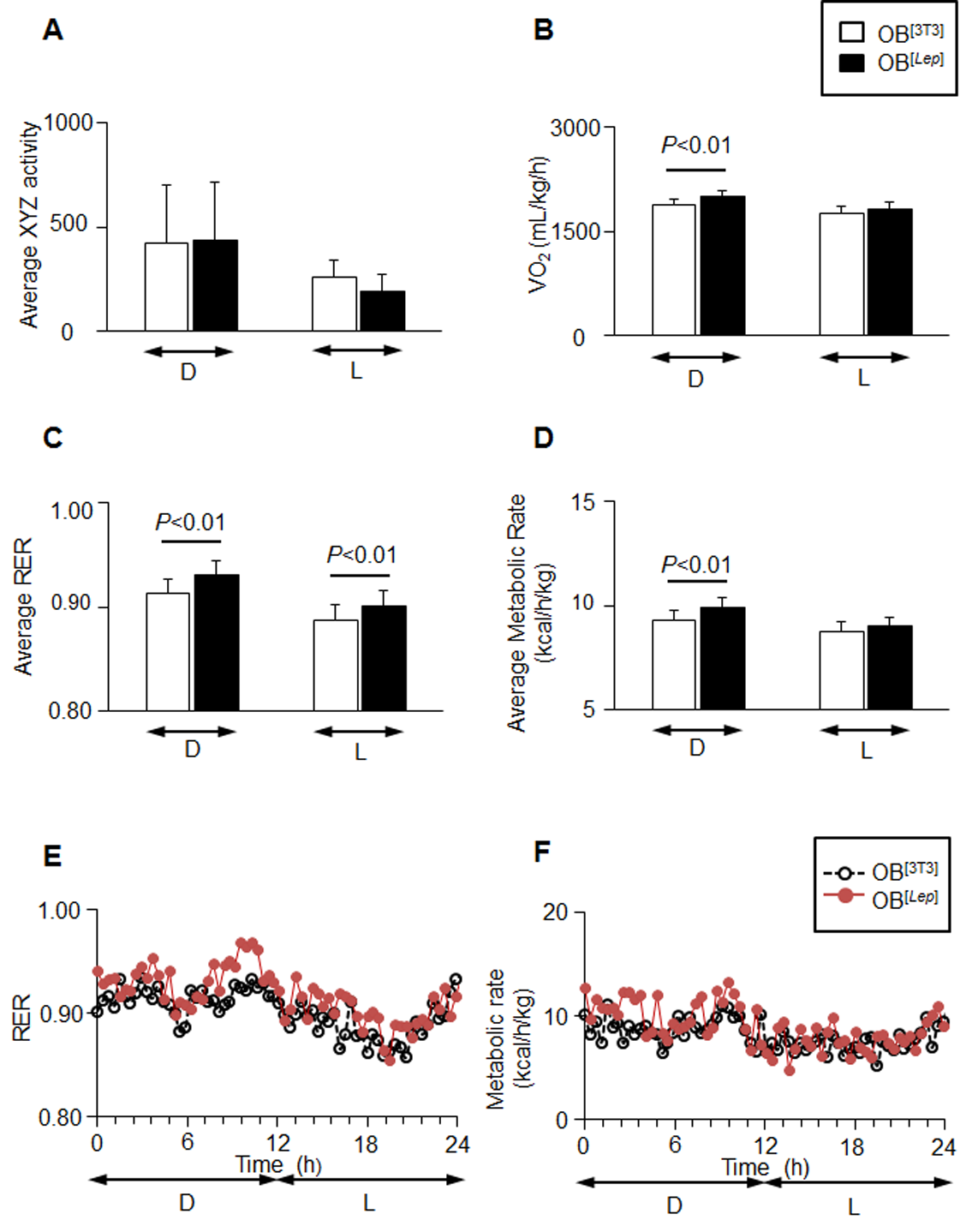

Supplement: S3 Fig — (A-D) Metabolic measurements were performed in OB[3T3] (white bar, n = 6) and OB[Lep] (black bar, n = 6) mouse groups in CLAMS metabolic cages at 28 days post injection. The average (mean ± SD) x, y, and z activity (A), average VO2 (mL/kg/h) (B), respiratory exchange ratio (RER) (C), and average metabolic rate (kcal/h/kg) (D) are shown for both the dark (D) and light (L) cycles. (E, F) Kinetic data for RER (E) and metabolic rate (kcal/h/kg) (F) are shown as mean for each time point in OB[3T3] (open black circles) and OB[Lep] (filled red circles) groups. (E, F) Kinetic data for RER (E) and metabolic rate (kcal/h/kg) (F) are shown as mean for each time point in OB[3T3] (open black circles) and OB[Lep] (filled red circles) groups. (TIF) [file pone.0153198.s003.tif]

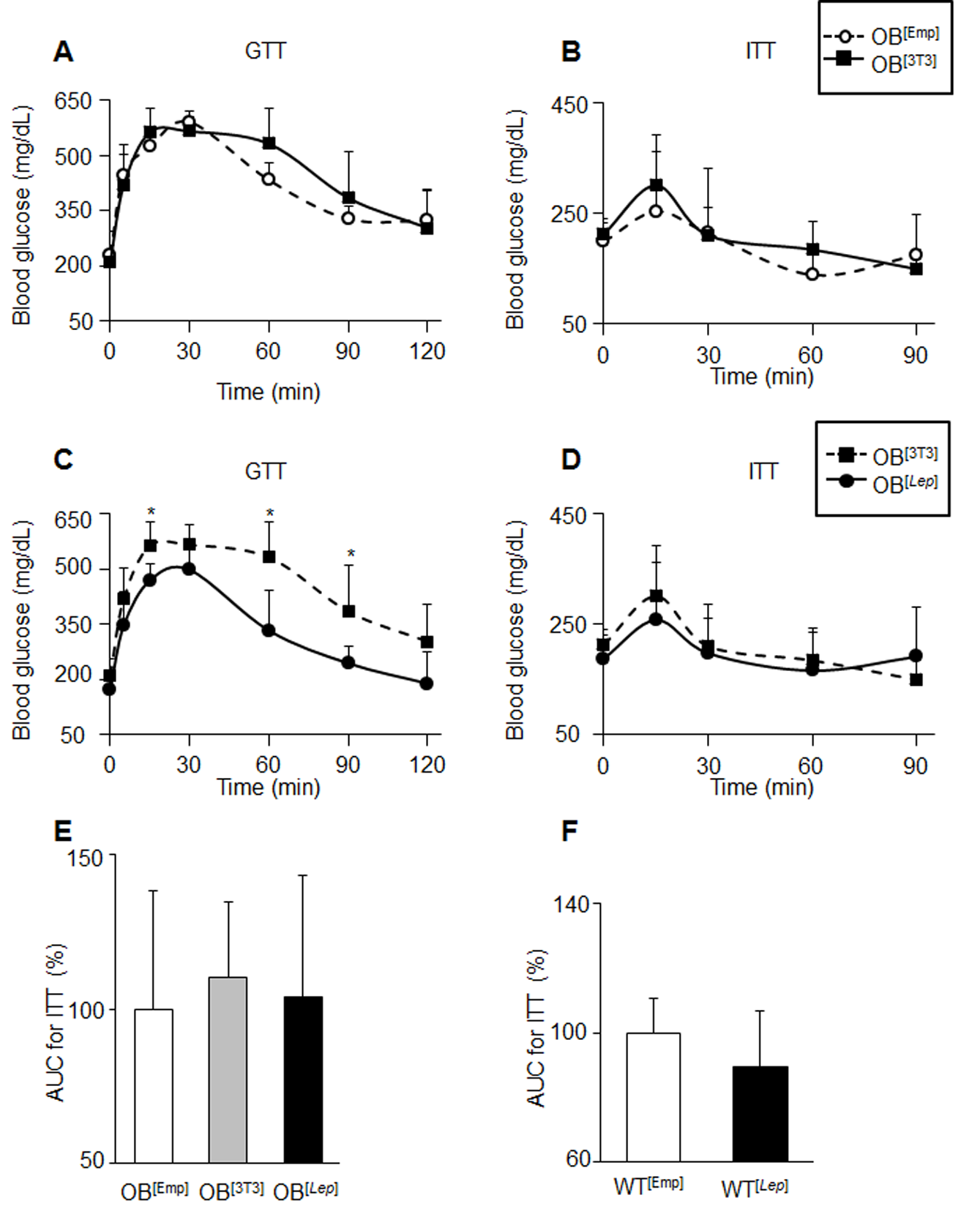

Supplement: S4 Fig — (A, B) Glucose tolerance tests (GTT) (A) or insulin tolerance tests (ITT) (B) were performed in fasting overnight mice OB[3T3] (open circles, n = 5) and OB[Lep] (filled circles, n = 5). Average blood glucose (mg/dL) are displayed for each time point. Asterisks indicate significant differences P<0.05, Student’s t-test. (C) The area under the curve (AUC) for each ITT was calculated using a trapezoidal approximation and displayed as percent of the control OB[Emp] (n = 5) group (100%). (D). WT mice with diet-induced obesity (Study 2) were injected with encapsulated acellular capsules (WT[Emp]) and encapsulated 3T3Lep (WT[Lep]) in both visceral fat pads (total 0.6*106 adipocytes for each cell type). ITT were performed in fasting overnight mice. Average AUC (mean ± SD) are shown for WT[Emp] (white bar, n = 3) and WT[Lep] (black bar, n = 5). (TIF) [file pone.0153198.s004.tif]
